# Scleroderma Renal Crisis: A Pathology Perspective

**DOI:** 10.1155/2010/543704

**Published:** 2010-07-28

**Authors:** Ibrahim Batal, Robyn T. Domsic, Thomas A. Medsger, Sheldon Bastacky

**Affiliations:** ^1^Department of Pathology, University of Pittsburgh Medical Center, A614 Scaife Hall, 200 Lothrop Street, Pittsburgh, PA 15213, USA; ^2^Division of Rheumatology and Clinical Immunology, University of Pittsburgh Medical Center, 3500 Terrace Street, BST, S7, Pittsburgh, PA 15261, USA

## Abstract

Scleroderma renal crisis (SRC) is an infrequent but serious complication of systemic sclerosis (SSc). It is associated with increased vascular permeability, activation of coagulation cascade, and renin secretion, which may lead to the acute renal failure typically associated with accelerated hypertension. The histologic picture of SRC is that of a thrombotic microangiopathy process with prominent small vessel involvement manifesting as myxoid intimal changes, thrombi, onion skin lesions, and/or fibrointimal sclerosis. Renal biopsies play an important role in confirming the clinical diagnosis, excluding overlapping/superimposed diseases that might lead to acute renal failure in SSc patients, helping to predict the clinical outcome and optimizing patient management. Kidney transplantation may be the only treatment option available for a subset of SRC patients who develop end-stage renal failure despite aggressive angiotensin-converting enzyme inhibitor therapy. However, the posttransplant outcome for SSc patients is currently suboptimal compared to the general renal transplant population.

## 1. Introduction

 Systemic sclerosis (SSc) is a multisystem autoimmune disorder that can manifest as either the diffuse cutaneous (dc) or the limited cutaneous (lc) variant, distinguished by the degree and the extent of cutaneous sclerosis [1]. Scleroderma renal crisis (SRC) can complicate the course of up to 10% of patients with SSc. Although most frequently seen in dcSSc, SRC can occur in patients with lcSSc [2, 3] and rarely in patients with no significant dermal sclerosis termed systemic sclerosis sine scleroderma (ssSSc) [4]. The etiology of SCR remains incompletely understood, with most models of pathogenesis suggesting an initial trigger of vascular endothelial injury. Alteration in cellular and/or humoral immunity may also play a role in SRC pathogenesis [1, 5, 6]. SSc has been associated with T helper lymphocyte type-2 (TH-2) activation, cytokine production (particularly Il-4, IL-13, and IL-17), and excess collagen accumulation, which could participate in the development of vasculopathy [7]. B cell activation has also been described in SSc patients [7]. The association between the presence of several specific autoantibodies and the development of SRC raises a potential contributing role of autoantibodies in the pathogenesis of SRC [8, 9]. In addition, antiendothelial cell antibodies, which are capable of inducing endothelial cell apoptosis [10] have been detected in up to 85% of SSc patients [11]. Overexpression of endothelin-1, a protein that plays a role in blood vessel constriction, and its receptor endothelin-B has been demonstrated in the small vessels of two SRC patients [12]. Furthermore, the C4d complement degradation product product is regarded as an immunologic marker of antibody-mediated rejection in renal allografts, has been detected in native renal biopsies from a subset of SRC patients [13]. 

 Subsequent to the potential endothelial triggering injury, the proposed cascade of histologic alterations is initiated by rapid increase in endothelial permeability and intimal edema. This then places the subendothelial connective tissue in direct contact with circulating blood elements activating the coagulation cascade and vascular thrombosis. The underlying connective tissue reacts to this insult by promoting fibroblastic and nonfibroblastic stromal proliferation, which manifests as proliferative endarteropathy (onion skin type lesion). Decreased renal perfusion as a result of arterial narrowing can additionally lead to juxtaglomerular apparatus (JGA) hyperplasia and renin secretion, resulting in accelerated hypertension and progressive renal injury. A milder form of vascular pathology, manifested usually as fibrointimal thickening can often be observed in SSc patients without SRC [14]. 

 Adequate renal biopsy specimens are generally capable of reflecting the aforementioned pathophysiologic changes. A detailed histologic assessment can confirm the clinical diagnosis and help exclude potential overlapping or superimposed etiologies.

## 2. Clinical and Laboratory Features

 SRC is typically characterized by a sudden and marked increase in systemic blood pressure (although normotensive SRC has been described [15]), and acute renal failure, with or without significant microangiopathic hemolytic anemia or thrombocytopenia. SRC is often accompanied by headache, blurring of vision, and dyspnea. These symptoms can be attributed to hypertensive encephalopathy, congestive heart failure, and/or pulmonary edema, respectively, as consequences of the rapid increase in blood pressure [16, 17]. Since SRC can present as acute renal failure, one would expect a significant elevation of serum creatinine and a considerable fall of glomerular filtration rate (GFR). In a relatively large study, the median serum creatinine value in SRC patients at presentation was 200 mmol/l (2.3 mg/dl) [18]. In our study, the median serum creatinine value at the time of biopsy was 362 mmol/l (4.1 mg/dl) [13]. 

 SRC more frequently affects females than males [1]. This may reflect the overall increased prevalence of SSc in the female population. Rapid progression of skin thickening in patients with SSc [19] and high doses of corticosteroid therapy [20] are risk factors for the development of SRC. The latter is usually associated with systemic steroid administration [20]. However, rare cases of SRC have been described following topical steroid use [21]. In patients with SRC, presenting normal or mildly elevated blood pressures (normotensive SRC), older age and male sex have been suggested to be adverse prognostic factors [18]. The poor prognosis in normotensive SRC patients might reflect ongoing subclinical renal injury leading to severe irreversible destruction of renal parenchyma due to delayed diagnosis. 

 Virtually all SRC patients have detectable antinuclear antibodies (ANA). Anti-RNA polymerase antibodies (especially types I and III), which are the most frequent autoantibodies encountered in North American dcSSc patients, are significantly associated with the development of SRC [8, 9, 22]. An association with antitopoisomerase I (anti-Scl 70) antibodies in dcSSc patients has also been reported. In contrast, anticentromere antibodies, which are commonly detected in lcSSc, are rarely encountered [19]. Anti-U3 RNP antibodies were found to have an association with SRC in some [23] but not all [24] studies. 

 Microangiopathic hemolytic anemia occurs in up to half of SRC patients [25] and is characterized by abrupt onset of anemia, the presence of schistocytes in the peripheral blood smear, and thrombocytopenia. Thrombotic microangiopathy is often accompanied by elevated serum LDH and decreased haptoglobin.

## 3. Gross Pathology

 Multiple, small petechial hemorrhages are frequently present on the surface of the affected kidneys. The cut section may reveal tiny wedge shaped infarcts and foci of cortical necrosis [26]. These changes are nonspecific and can be observed in other thrombotic microangiopathic disorders, such as hemolytic uremic syndrome, thrombotic thrombocytopenic purpura, and idiopathic malignant hypertension, or in association with some medications.

## 4. Microscopic Pathology

 Renal biopsies, even though necessary to confirm the diagnosis, are not routinely warranted in SRC. Theoretically, unless the patient is suffering from typical clinical features and is associated with thrombotic microangiopathy picture on peripheral blood examination, the diagnosis cannot be confirmed with certainty without a renal biopsy. However, renal biopsy is an invasive procedure. Practically, such biopsies are recommended when doubt exists about the etiology of renal dysfunction, or, alternatively, to exclude the presence of other pathologic conditions.

 The overall microscopic picture is that of a thrombotic microangiopathic process [26, 27]. Similar to idiopathic malignant hypertension, and in contrast to hemolytic uremic syndrome and thrombotic thrombocytopenic purpura, primary small vessel changes usually predominate over glomerular alterations in SRC. Small vessel thrombi outnumbered glomerular thrombi in SRC [11/17 (65%) versus 3/17 (18%), *P* = .01] [13], while the opposite was found in hemolytic uremic syndrome; thrombotic microangiopathy changes were more commonly detected in the glomeruli compared to small vessels [11/12 (92%) versus 4/12 (33%), *P* = .009] [28]. 

**Figure 1:**
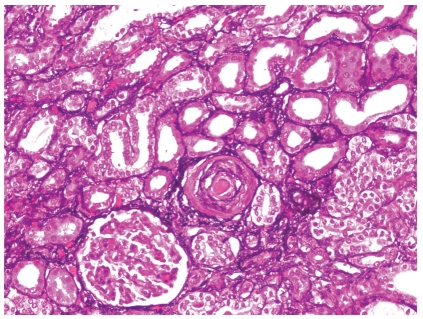
Arterial thrombosis associated with prominent glomerular ischemic collapse in a patient with scleroderma renal crisis (Methenamine silver stain; original magnification x100).

**Figure 2:**
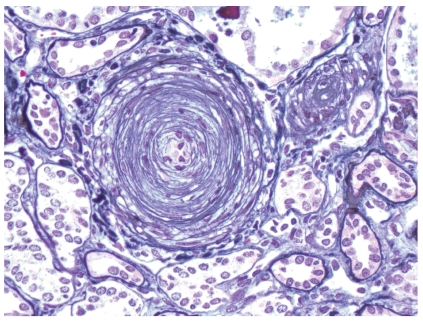
Prominent arterial onion skin lesion in a patient with scleroderma renal crisis. Such lesions often cause severe vascular narrowing leaving only a pinpoint open lumen (Methenamine silver stain; original magnification x400).

**Figure 3:**
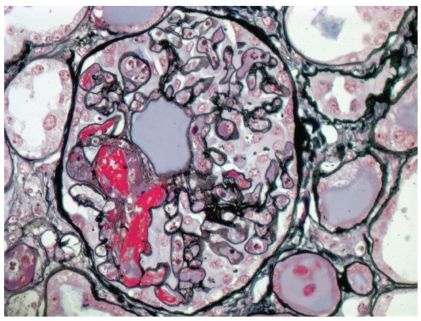
Glomerular capillary thrombosis in a patient with scleroderma renal crisis. This finding is rather infrequent in scleroderma renal crisis and is more commonly observed in hemolytic uremic anemia and thrombotic thrombocytopenic purpura (Methenamine silver stain; original magnification x600).

**Figure 4:**
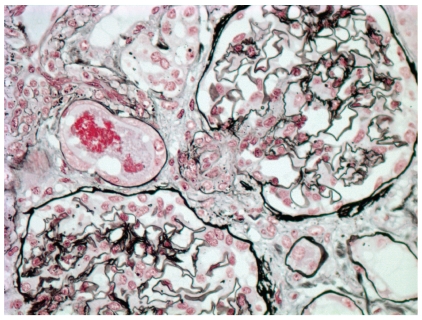
Prominent juxtaglomerular apparatus containing sparse silver positive renin granules in a patient with scleroderma renal crisis (Methenamine silver stain; original magnification x400).

 Histologic manifestations may vary during the course of the disease. Early vascular changes can manifest as intimal accumulation of myxoid material, thrombosis (Figure 1), and/or fibrinoid necrosis. Onion-skin lesions develop later (Figure 2), while fibrointimal sclerosis with or without adventitial fibrosis may be the only manifestation of chronic ongoing damage or organization resulting from previous episodes of acute injury. Acute glomerular changes can occur primarily or often develop secondary to the vascular injury and reduction in renal perfusion. Primary glomerular changes appear to be related to glomerular endothelial injury. These can manifest as endothelial swelling and glomerular capillary thrombosis (Figure 3). The latter is relatively infrequent [13]. Chronic glomerular changes, which include basement membrane double contours (tram tracking) and glomerulosclerosis, may develop later. Secondary glomerular changes may result in ischemic glomerular collapse. JGA hyperplasia, a histologic sequel of increased renin production can be observed microscopically (Figure 4). Prominent JGA hyperplasia was found to be present in 2/17 (12%) of our SRC cases [13]. Tubulointerstitial changes, which are also secondary to vascular pathology, are frequently manifested as ischemic acute tubular injury/necrosis or, if more chronic, as tubular atrophy and interstitial fibrosis. A lymphohistiocytic interstitial inflammatory infiltrate can occasionally be observed.

 Finally, even though SRC represents an acute form of renal involvement, vascular pathology may be observed in SSc patients in the absence of SRC. Trostle et al. [14], in a case control autopsy study, compared the intimal surface areas of renal arteries in SSc autopsy cases (SRC, dcSSc without SRC, and lcSSc without SRC) to age- and sex-matched autopsy controls. Using morphometric techniques, these investigators confirmed that SRC patients had a significant increase in renal arterial intimal thickening. Interestingly, they also found that, in the absence of SRC, a significant increase in arterial fibrointimal thickness was observed in dcSSc patients, and to a lesser extent in lcSSc patients, compared to controls. Such vascular changes may be due to the presence of mild ongoing renal vascular injury below the threshold which triggers SRC.

## 5. Ancillary Studies

 Immunofluorescence and electron microscopy are routinely used ancillary studies for evaluating native renal biopsies. Immunofluorescence studies are mainly utilized to characterize the presence, nature, pattern of staining, and anatomic distribution of immune deposits. Electron microscopy is used for ultrastructural assessment of renal biopsies. It is extremely helpful in localizing and characterizing immune complex and protein deposits with organized substructures (amyloidosis, fibrillary etc.) and to assess the glomerular endothelium, basement membrane, and podocytes. 

 Routine ancillary studies are of limited value in confirming the diagnosis of SRC. There are only a few reports which have characterized the immunofluorescence findings in SRC. Immunoglobulin deposits in the glomeruli and/or blood vessels were identified in most but not all of these studies. Among the immunoglobulin deposits, IgM, which might be considered the result of a nonspecific entrapment, was the most frequently detected [29–31]. This was often accompanied by complement deposits. 

**Figure 5:**
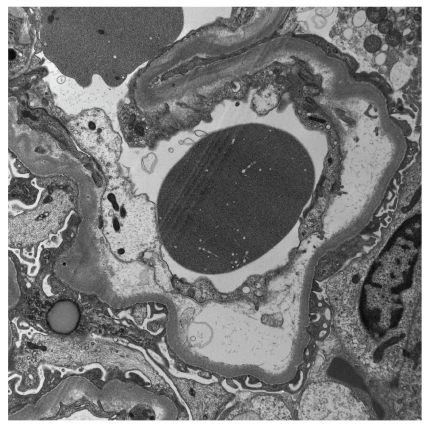
Electron microscopy from a patient with scleroderma renal crisis reveals detachment of the endothelium and prominent electron lucent fluffy material (Electron microscopy; original magnification x5600).

 In SRC, electron microscopic evaluation frequently fails to detect discrete electron dense deposits. Hyaline material often accumulates in the subendothelium of the glomeruli and/or blood vessels in SRC [29–32]. Of note, hyaline deposits can sometimes be difficult to distinguish from definite immune complex deposits. Evidence of endothelial injury such as endothelial swelling and prominent accumulation of glomerular subendothelial electron lucent material have been described in SRC (Figure 5) but also in malignant hypertension [33]. Myointimal cells were detected in the extended fibrointima in SRC patients [34].

 C4d is a complement split product which is generated following complement activation via classical or mannose-bound lectin pathways. C4d can be detected using immunoperoxidase on formalin fixed tissue or immunofluorescence on frozen tissue. The former is technically easier to perform while the latter is considered slightly more sensitive and specific. We identified finely granular C4d staining in the peritubular capillary of a subset of SRC patients who had associated poor renal outcome [13]. In allograft kidneys, the detection of peritubular capillary C4d staining is usually associated with antibody-mediated rejection and poor allograft outcome [35–37]. Confocal immunofluorescence can potentially play an important role in localizing C4d as a target. Although a larger multicenter study using immunofluorescence technique is needed to validate our preliminary findings and to further characterize the cause of such deposits, evidence supporting the role of antibody-mediated injury in SSc/SRC patients is accumulating. First, disease-specific serum autoantibodies have been found to be associated with certain clinical manifestations [22]. Second, antiendothelial antibodies and increased expression of endothelin-1/endothelin-B have been detected in a subset of SRC patients [12]; it is noteworthy that overexpression of endothelin-1 gene has been recently discovered in allograft kidneys with antibody-mediated rejection [38]. Lastly, antiglobulin antibodies have been found in the eluate of several SRC kidney samples [30].

## 6. Differential Diagnosis

 Clinically, SRC should be suspected when acute renal failure (ARF) develops in SSc patients. Nevertheless, ARF occurring in SSc patients is not always due to SRC. Renal artery stenosis, hypovolemia, crescentic glomerulonephritis (GN), and other renal diseases may also occur in SSc patients [27]. These disorders may result in a similar clinical picture. A thrombotic microangiopathy-like clinical picture can even be encountered in patients with renal arterial stenosis [27]. Distinguish SRC from crescentic GN is critical since immunosuppressive therapy is used to treat the latter. As the name implies, the presence of crescents is the hallmark of crescentic GN. In typical SRC cases, crescents are extremely rare and, when detected, are very small [39]. In SSc patients, most of the encountered crescentic GN are ANCA-associated. These are pauci-immune on immunofluorescence studies, associated with anti-myeloperoxidase antibodies, and usually triggered by penicillamine [40, 41]. Less often, one may encounter immune complex GN [39] or antiglomerular basement membrane GN [42]. Immunofluorescence studies reveal granular glomerular basement membrane and/or mesangial immune complex deposits in the former and linear glomerular basement membrane IgG staining in the latter.

**Figure 6:**
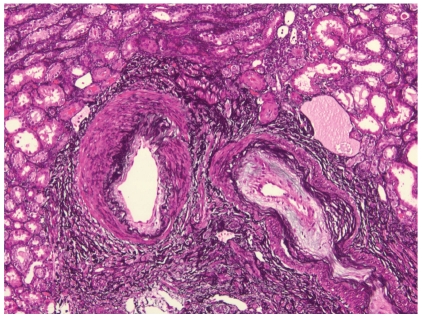
Prominent arterial adventitial fibrosis in a patient with scleroderma renal crisis. Note that the arteries also have mild intimal accumulation of myxoid material. (Methenamine silver stain; original magnification x100).

 Histologically, thrombotic microangiopathic changes can be observed in several disorders. Although it is often impossible to ascertain the specific cause of thrombotic microangiopathy based on histologic evaluation alone, it is important to note that extraglomerular small vessel vascular lesions often predominate in SRC while primary glomerular capillary microangiopathic changes (glomerular capillary thrombosis) are a relatively infrequent histologic finding in SRC [13]. The presence of JGA hyperplasia has been described in SRC patients [43, 44]. Similarly, vascular adventitial fibrosis (Figure 6) [45] has been regarded by some investigators to be characteristic for SSc [46] and SRC [47]. Clinicopathological correlation, however, is often required to achieve the correct diagnosis. In advanced stages of SRC, the histologic findings are usually nonspecific and reflect advanced chronic renal damage. At such late stages, it is often difficult to distinguish chronic vascular changes associated with organized SRC from preexisting chronic accelerated essential hypertension. The presence of peri-adventitial fibrosis, if prominent, might be helpful as a point in favor of SRC.

## 7. Prognosis

 Several retrospective studies have investigated the role of renal biopsy in predicting prognosis in SRC. Penn et al. showed that the presence of acute vascular changes (myxoid intimal thickening and thrombosis) were associated with poor prognosis [18]. We showed that the severity and extent of acute vascular injury, including fibrinoid changes and/or thrombosis, was most predictive of poor outcome [13]. We also showed that severe glomerular ischemic collapse, and to a lesser extent acute tubular necrosis, may also be associated with poor prognosis [13]. Both of the latter are secondary changes reflecting the severity of vascular lesions. In contrast to acute changes, we observed that chronic renal changes did not significantly correlate with poor outcome [13]. A recently published abstract suggested that chronic pathological changes might be associated with a favorable prognosis [48]. The latter observation is difficult to explain since chronic changes are typically irreversible and are expected, if any, to have an adverse impact on renal survival, as was described in other kidney diseases such as lupus nephritis [49].

## 8. Treatment/Outcome

 Blood pressure should be vigorously and aggressively controlled in patients with established SRC. The mortality associated with SRC has significantly decreased due to early diagnosis and aggressive angiotensin-converting enzyme (ACE) inhibitor therapy [50, 51]. Still, a subset of SRC patients may be refractory to ACE-inhibitor and other hypertensive therapy. These patients often remain on dialysis or die [17, 51, 52]. Kidney transplantation should be considered if the condition does not reverse despite aggressive treatment (usually within two years) [53].

## 9. Posttransplant Outcome

**Figure 7:**
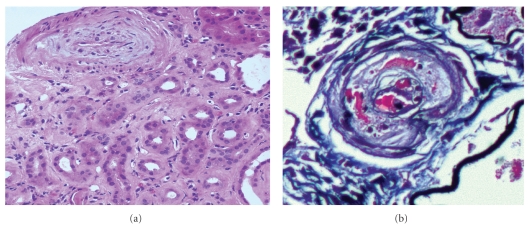
Two renal allograft biopsies with histologic features suspicious for recurrence of scleroderma. Note the prominent myxoid changes in the artery in biopsy (a) as well as the severe intimal thickening of blood vessels, which is accompanied by thrombosis and schistocytes within the arteriole wall in biopsy (b). The differential diagnosis includes acute antibody-mediated rejection and acute calcineurin inhibitor toxicity. Clinical correlation with the presence of C4d stain, detection of circulating donor-specific antibodies, and calcineurin inhibitor levels are usually warranted to achieve a correct diagnosis [(a) H&E; original magnification x200 and (b) Methenamine silver stain; original magnification x400].

 Although renal transplantation offers superior survival in SRC patients, graft survival is frequently reduced in SSc-induced renal failure compared to the general renal transplant population [54, 55]. We retrospectively studied the posttransplantation course of 10 SRC patients [56]. One, three, and five year graft survivals in this SRC cohort of patients were 70%, 70%, and 25%, respectively, compared with approximately 90%, 79%, and 75% graft survival in miscellaneous patients who received kidney transplants from deceased donors at the same institution [57]. Recurrence of scleroderma (Figure 7) may play a role in this poor post-renal transplant outcome [58, 59]. Two of our 10 patients had histologic features suspicious for SRC recurrence, manifested by both exacerbated development of arterial fibrointimal thickening with thrombotic microangiopathy-like changes [56]. Pham et al. [59] found that recurrent SRC occurs early in the course of transplantation (within 2 years post-transplantation). However, Cheung et al. [58] challenged the conventional experience by reporting a recurrence of SRC which occurred seven years post-transplantation. This SRC occurred following switching therapy from an ACE inhibitor to an angiotensin II receptor blocker. 

 Establishing a histologic diagnosis of recurrent scleroderma/SRC in an allograft is more challenging than diagnosing SRC in a native kidney biopsy. In addition to recurrent SRC, the pathologic differential diagnosis in allograft biopsies with a thrombotic microangiopathy-like picture also includes antibody-mediated rejection, calcineurin inhibitor toxicity, infection, and other less common allograft-related abnormalities [60]. More chronic changes can also be difficult to distinguish from de novo transplant glomerulopathy.

 In summary, SRC is a severe complication of systemic sclerosis. Although not always clinically warranted, renal biopsy can play an important role in establishing the diagnosis and in excluding other pathologic conditions such as vasculitis and connective tissue disease related and nonrelated syndromes. Furthermore, renal biopsies can help to predict renal prognosis and may contribute to our better understanding of the mechanisms and pathologic manifestations of SRC, ultimately leading to optimization of treatment strategies.
